# Trends in orphan medicinal products approvals in the European Union between 2010–2022

**DOI:** 10.1186/s13023-024-03095-z

**Published:** 2024-02-27

**Authors:** Luísa Bouwman, Bruno Sepodes, Hubert Leufkens, Carla Torre

**Affiliations:** 1 Faculdade de Farmácia, Universidade de Lisboa, Lisbon, Portugal; 2 Laboratory of Systems Integration Pharmacology, Clinical and Regulatory Science, Research Institute for Medicines of the University of Lisbon (iMED.ULisboa), Lisbon, Portugal; 3 Division of Pharmacoepidemiology and Clinical Pharmacology, Utrecht Institute for Pharmaceutical Sciences (UIPS), Utrecht University, Utrecht, The Netherlands

**Keywords:** Orphan regulation, Rare diseases, Orphan medicines, Accelerated assessment, Incentives

## Abstract

**Background:**

Over the last twenty years of orphan drug regulation in Europe, the regulatory framework has increased its complexity, with different regulatory paths and tools engineered to facilitate the innovation and accelerate approvals. Recently, the proposal of the new Pharmaceutical Legislation for the European Union, which will replace at least three Regulations and one Directive, was released and its new framework is raising many questions. The aim of this study was to present a characterisation of the Orphan Medicinal Products (OMPs) authorised by the European Commission (EC), between 2010 and 2022, looking into eighteen variables, contributing to the ongoing discussion on the proposal and implementation of the new Pharmaceutical Legislation proposed.

**Methods:**

Data of the OMPs identified and approved between 2010 and 2022 were extracted from the European Public Assessment Reports (EPARs) produced by the European Medicines Agency. Information regarding legal basis of the application, applicant, protocol assistance received, type of authorization, registration status, type of molecule, ATC code, therapeutic area, target age, disease prevalence, number of pivotal clinical trials supporting the application, clinical trial designs, respective efficacy endpoints and number of patients enrolled in the pivotal clinical trials were extracted. A descriptive statistical analysis was applied.

**Results:**

We identified 192 OMPs approved in the period between 2010 and 2022. 89% of the OMPs have legal basis of “full application”. 86% of the sponsors received protocol assistance whereas 64% of the MAA benefited from the accelerated assessment. 53% of the active substances are small molecules; about 1 in 5 molecules are repurposed. 40% of the OMPs have oncological therapeutic indications and 56% of the OMPs are intended to treat only adults. 71% of the products were approved based on a single pivotal trial.

**Conclusions:**

This analysis of OMPs approved between 2010 and 2022 shows that a shift has occurred in the rare disease medicine development space. Through the period studied we observe an increase of non-small molecules approved, accelerated assessment received and non-standard MA’s granted.

## Background

Orphan medicinal products (OMPs) are medicines intended to treat, prevent, or diagnose a disease that is life-threatening or chronically debilitating and for which the prevalence of the condition, in the European Union (EU), is not more than 5 in 10,000 [1]. The orphan legislation came into force in EU in 2000, with the promulgation of Regulation (EC) No 141/2000, addressing the need to offer incentives for the development and marketing of medicines for rare conditions [1]. These rare diseases are a group of an estimated 6,000 to 9,000 different conditions that affect more than 30 million people in Europe [2]. About 95% of rare diseases do not have yet any treatment approved [3].

Over the last two decades, the regulatory processes, guidance, and recommendations at the European Medicines Agency (EMA) have evolved and, at the same time, increased in complexity considering that drug development itself is more complex with the advancements in science being translated into new medicines. Additional programs and regulatory tools have been created to accelerate drug approval of medicines addressing unmet medical needs. Some of these tools are the possibility of accelerated assessment, the priority medicines (PRIME) program, and the conditional approval or the authorization under exceptional circumstances [4–9].

There are some studies published analysing the OMPs approved regarding the type of application, type of molecule, target diseases, and assessing the methodological quality of the OPMs dossiers, either in EU or in United States (US) [10–14]. Here we present, to our knowledge, for the first time, an extensive characterisation of OMPs approved in the EU, considering eighteen criteria for the last thirteen-year period. Moreover, the findings of this study can raise important questions and provide learnings that may be relevant to the on-going reflection on the upcoming revised EU legislation, which could be the largest reform in over 20 years in this area.

Therefore, the aim of this study was to identify and analyse the OMPs approved by the European Commission (EC), between 2010 and 2022, which can be of value for various stakeholders, including regulators, drug developers, academia and European legislators involved in implementation of the new pharmaceutical legislation.

## Methods

The publicly available search-database of the EMA was used to identify all products approved in EU through the centralised procedure between January 2010 and December 2022, including those that were subsequently withdrawn. European Public Assessment Reports (EPARs) of the identified OMPs were retrieved from the EMA’s website. We included products with orphan drug designation at the time of Committee for Human Medicinal Products (CHMP) opinion, even if the applicant requested the removal of the orphan drug designation at the time of the MA granting.

The following data were extracted from the EPARs: year of approval, registration status, legal basis of the application, applicant’s company name, protocol assistance received (yes/no), type of molecule (small molecule, biological, advanced therapy medicinal product—ATMP—or oligonucleotide), ATC code, therapeutic area, target age, disease prevalence, repurposing (yes/no), accelerated assessment (yes/no), additional monitoring (yes/no), type of MA granted (standard, conditional or under exceptional circumstances), number of “pivotal” or”main” efficacy clinical trials, clinical trial design, type of primary efficacy endpoints (clinical, functional or surrogate marker) and total number of patients included or randomized (in case of randomized controlled trials—RCT), for each indication, in the pivotal clinical trials. In case of randomized controlled trials, we included in this analysis all patients randomized i.e., intention-to-treat population (ITT). In case of open label studies, we included all patients enrolled in the mentioned open label studies identified as “main” or “pivotal” studies.

Small molecules are a type of a chemical substance defined by a single molecular structure that is not a protein or nucleic acid substance. Biologicals are defined by the European legislation as ‘a medicine that contains one or more active substances made by or derived from a biological source’ (Article 4(7) of Regulation (EU) 2019/6 of 11 December 2018). Oligonucleotides are at the interface of small molecules and biologicals. Oligonucleotides are short, single- or double-stranded DNA or RNA molecules and include antisense oligonucleotides, RNA interference and aptamer RNAs. Advanced Therapy Medicinal Products (ATMPs) are any of the following medicinal products for human use: a gene therapy medicinal product as defined in Part IV of Annex I to Directive 2001/83/EC, a somatic cell therapy medicinal product as defined in Part IV of Annex I to Directive 2001/83/EC, a tissue engineered product that contains or consists of engineered cells or tissues and is presented as having properties for, or is used in or administered to human beings with a view to regenerating, repairing or replacing a human tissue, as defined by article 2 of Regulation (EC) No 1394/2007 of 13 November 2007.

The micro, small and medium-sized enterprises (SMEs) with an official SME status are registered in a publicly available register of SMEs at the EMA website. This register includes companies established in the European Economic Area that have submitted a SME declaration within the meaning of Recommendation 2003/361/EC and to whom the Agency has assigned SME status.

We considered the following age categories: a) children (including babies of different ages until 11 years old); b) children and adolescents (patients with age between 12 and 17 years old); c) adults and adolescents; d) adults and children; e) adults; f) adults and elderly; g) elderly and h) all ages.

The prevalence of the disease reported in the orphan drug designation (ODD) report was cross-checked with the prevalence mentioned in the EPAR and in the Orphan maintenance assessment report (OMAR), in place since 2018, and available at the EMA website. In case different prevalences are reported in these documents, we included the most recent one, i.e. the prevalence mentioned in the OMAR.

A medicine is defined “repurposed” when the active substance is currently used in clinical practice for a new indication, outside the scope of the original medical indication as defined by *Langedijk *et al. [15].

The data extracted from the EPARs were used to build an Excel database for all OMPs approved by the EC between 2010 and 2022. This information was cross-checked with the annual reports available at EMA website. A descriptive statistical analysis was applied.

## Results

We identified 192 OMPs approved in the period between 2010 and 2022.

Table 1 presents the results obtained for the variables studied.

**Table 1:** Characteristics of orphan medicinal products approved between 2010 and 2022

Variable	No of OMPs (n = 192)	Percentage (%)
**Year of approval by the European Commission**		
2010	5	2.6
2011	4	2.1
2012	10	5.2
2013	7	3.6
2014	14	7.3
2015	18	9.4
2016	18	9.4
2017	17	8.9
2018	24	12.5
2019	7	3.6
2020	22	11.5
2021	20	10.4
2022	26	13.5
**Legal basis of the MA application**		
Article 8.3	171	89.1
Article 10.3	12	6.3
Article 10(a)	6	3.1
Type II variation	2	1.0
Article 10(b)	1	0.5
**Registration status (signoff date: 31 dec 2023)**		
Approved	186	96.9
Withdrawn	6	3.1
**SME status**		
Yes	19	9.9
No	173	90.1
**Protocol assistance**		
Yes	165	85.9
No	27	14.1
**Type of MA**		
Standard	129	67.2
Conditional	41	21.4
Exceptional circumstances	22	11.5
**Accelerated assessment**		
Yes	124	64.6
No	68	35.4
**Type of molecule**		
Small molecule	101	52.6
Biologic	67	34.9
ATMP	15	7.8
Oligonucleotide	6	3.1
Herbal preparation	2	1.0
Radiopharmaceutical	1	0.5
**Repurposing**		
No	159	82.8
Yes	33	17.2
**ATC Code**		
L Antineoplastic and immunomodulating agents	76	39.6
A Alimentary tract and metabolism	33	17.2
B Blood and blood forming organs	21	10.9
J Antiinfectives for systemic use	12	6.3
N Nervous system	11	5.7
H Systemic hormonal preparations, excluding sex hormones and insulins	9	4.7
M Musculo-skeletal system	6	3.1
C Cardiovascular system	5	2.6
R Respiratory system	5	2.6
S Sensory organs	5	2.6
D Dermatologicals	3	1.6
V Various	2	1.0
G Genito-urinary system and sex hormones	1	0.5
P Antiparasitic products, insecticides, and repellents	1	0.5
Not assigned yet	2	1.0
**Therapeutic areas (TOP 5 oncological diseases)**		
1 Multiple Myeloma	12	6.3
2 Leukemia Myeloid	8	4.2
3 Lymphoma’s	8	4.2
4 Leukemia non-Myeloid	7	3.6
5 Lymphoblastic Leukemia-Lymphoma	4	2.1
**Therapeutic areas (TOP 5 genetic diseases)**		
1 Haemophilia (A and B)	7	3.6
2 Cystic fibrosis	6	3.1
3 Muscular Dystrophy or Atrophy	5	2.6
4 Familial Amyloidosis	4	2.1
5 Anemias	4	2.1
**Therapeutic areas (TOP 1 infections)**		
1 Tuberculosis	4	2.1
**Target age**		
Adults	107	55.7
Adults and children	40	20.8
Adults and adolescents	21	10.9
All ages	12	6.3
Children	6	3.1
Adolescents and children	4	2.1
Adults and elderly	1	0.5
Elderly	1	0.5
**Disease prevalence (per 10,000)**		
< 0.2 (ultra rare)	41	19.9
0.2–0.5	27	13.1
0.6– ≤ 1	40	19.4
> 1– ≤ 2	36	17.5
> 2– ≤ 4	50	24.3
> 4–5	12	5.8
**Additional monitoring**		
Yes	124	64.6
No	68	35.4
**Number of pivotal efficacy clinical trials**		
No clinical studies performed	10	5.2
1	136	70.8
2	39	20.3
3	4	2.1
4	2	1.0
5	1	0.5
**Main clinical trial designs (n = 241)**		
Randomized, double blind, controlled trial	117	48.5
Randomized, single blind	2	0.8
Randomized, open label	42	17.4
Partially randomized, open label	1	0.4
Non-randomized, open label, sequential	1	0.4
Open label, single arm	65	27.0
Open label, 2-arm	3	1.2
Open label, 4-arm	2	0.8
Retrospective studies	5	2.1
Observational cohorts	3	1.2
**Type of primary efficacy endpoints (n = 181)**		
*Oncological OMPs (n = 66)*		
Surrogate	52	78.8
Clinical	14	21.2
*OMPs addressing genetic diseases (n = 109)*		
Clinical	44	40.4
Surrogate	37	33.9
Functional	28	25.7
*Anti-infectives OMPs (n = 6)*		
Clinical	2	33.3
Surrogate	4	66.7

### Removal of the ODD upon MAH request

**Fig. 1:**
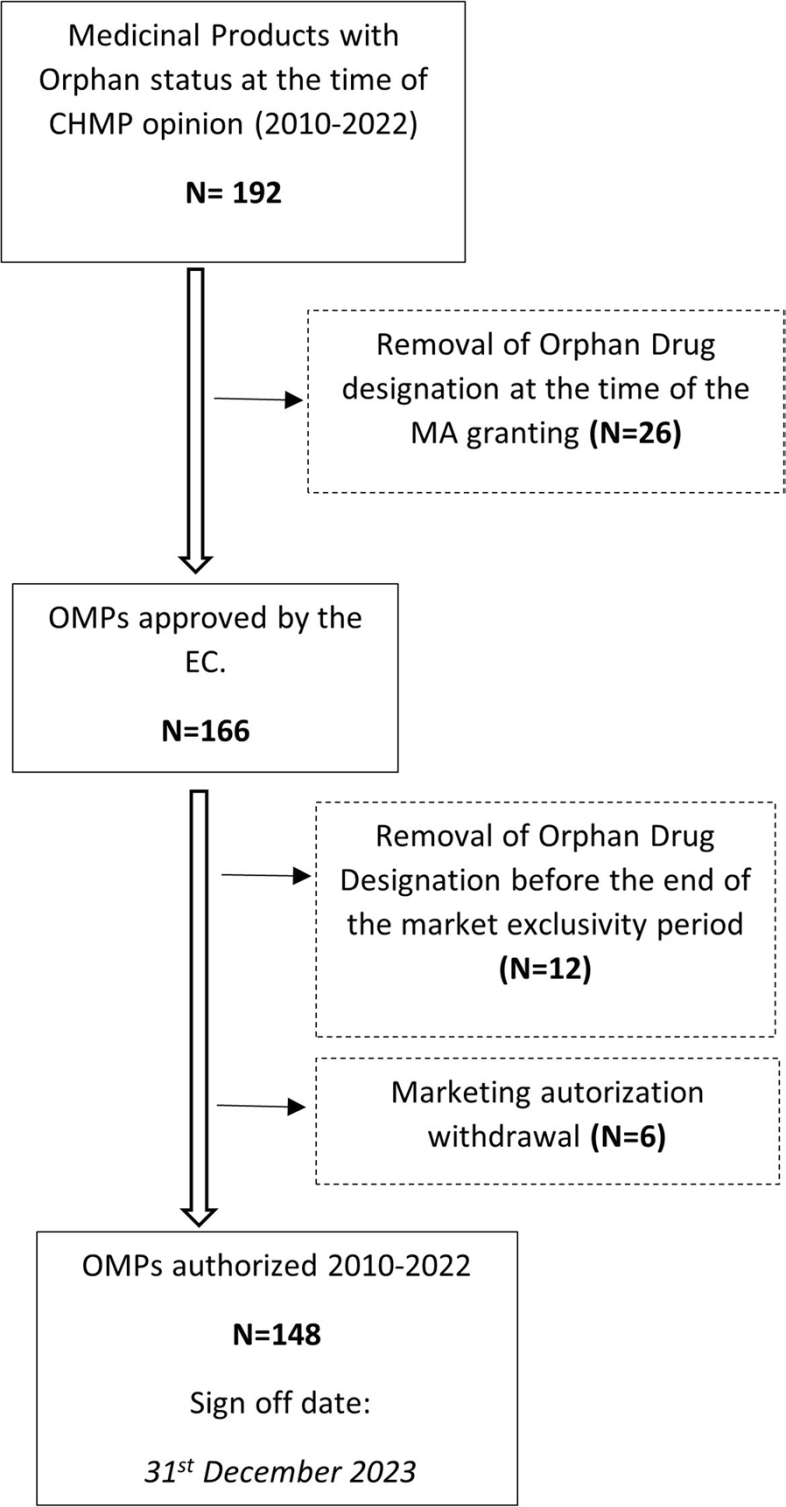
Retrieval process (Sign off date: 31st December 2023)

Almost one fifth (n = 37; 19%) of marketing authorization holders (MAH) requested the withdrawal of the ODD. Most of these withdrawals occurred at the time of the granting of the MA (n = 25), after the Committee for Orphan Medicinal Products’s (COMP) reviewed of the orphan designation criteria at the time of marketing authorisation. We have also identified OMPs for which the ODD was removed (n = 12), by the MAH, during the market exclusivity period. The following figure (Fig. 1) schematizes this process.

In the group of medicines for which the ODD was removed at the time of the MA granting, 9 of the 25 OMPs have ATC code L01—Antineoplastic agents. Regarding the reason for the ODD withdrawal, we only have data available since 2018, i.e., 11 OMPs. Among these, for 6 OMPs the reasons were a combination of further clarification needed regarding the prevalence calculations and significant benefit not proved; in the other 5 OMPs the reason was lack of clinical data proving the significant benefit.

In the group of medicines for which the ODD was removed before the end of the market exclusivity period, 10 out of the 12 OMPs have ATC code L01- Antineoplastic agents. No justification or report was available in these cases regarding the potential reasons for this premature withdrawal.

### Registration status

Of the 192 OMPs identified, 6 are no longer authorised (sign off date: 31 December 2023). Of these 6 withdrawn products, 4 (67%) had received a conditional approval, one (17%) was approved under exceptional circumstances, and one (17%) had a standard MA. Regarding the type of molecule, 3 (50%) are biologicals and 3 (50%) are ATMPs. The reasons for these withdrawals mentioned in the public withdrawal letters are in 5 out of the 6 (83%) OMPs commercial reasons and in one case (17%) it was due to a referral. Olaratumab was indicated to treat soft tissue sarcoma in combination with doxorubicin. However, after analysing the final results of the phase 3 study the EMA concluded that olaratumab did not prolong the survival in the overall population and therefore recommended the withdrawal of the MA.

### Legal basis of the application

Of the 192 OMPs identified, 171 (89%) have legal basis Article 8.3—full application, 12 (6.3%) legal basis 10.3—hybrid application, 6 (3%) legal basis 10(a)—well established use. Two (1%) were approved through a type II variation and 1 (0.5%) was approved as 10(b) fixed-combination application (Table 1).

### SME status

We identified 121 different applicants in the period studied. Only 17 (14%) of these applicants have currently a SME status in the online SME register (sign off date: September 2023). One of these applicants has submitted two applications in the period studied. Therefore, we identified in total 19 OMPs (9.9%) for which the applicant still was a SME. In fact, the companies with more MA’s are large multinational companies.

### Protocol assistance

According to the information presented in the EPARs, 165 of the 192 OMPs (86%) received protocol assistance.

### Accelerated assessment

**Fig. 2:**
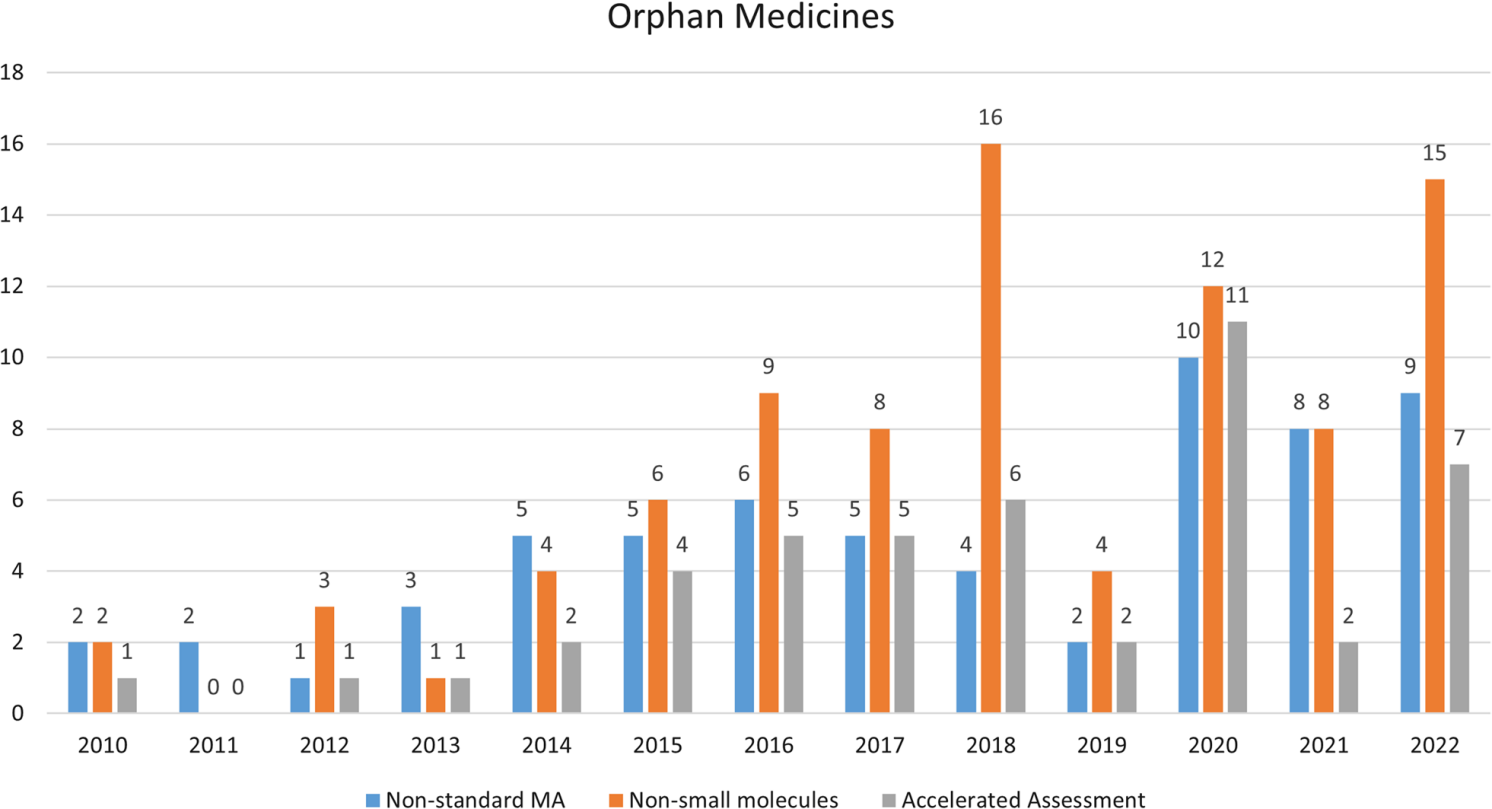
Evolution of the number of OMPs which have received a non-standard marketing authorization, OPMs which are non-small molecules, and OMPs which have benefited from accelerated assessment

**Fig. 3:**
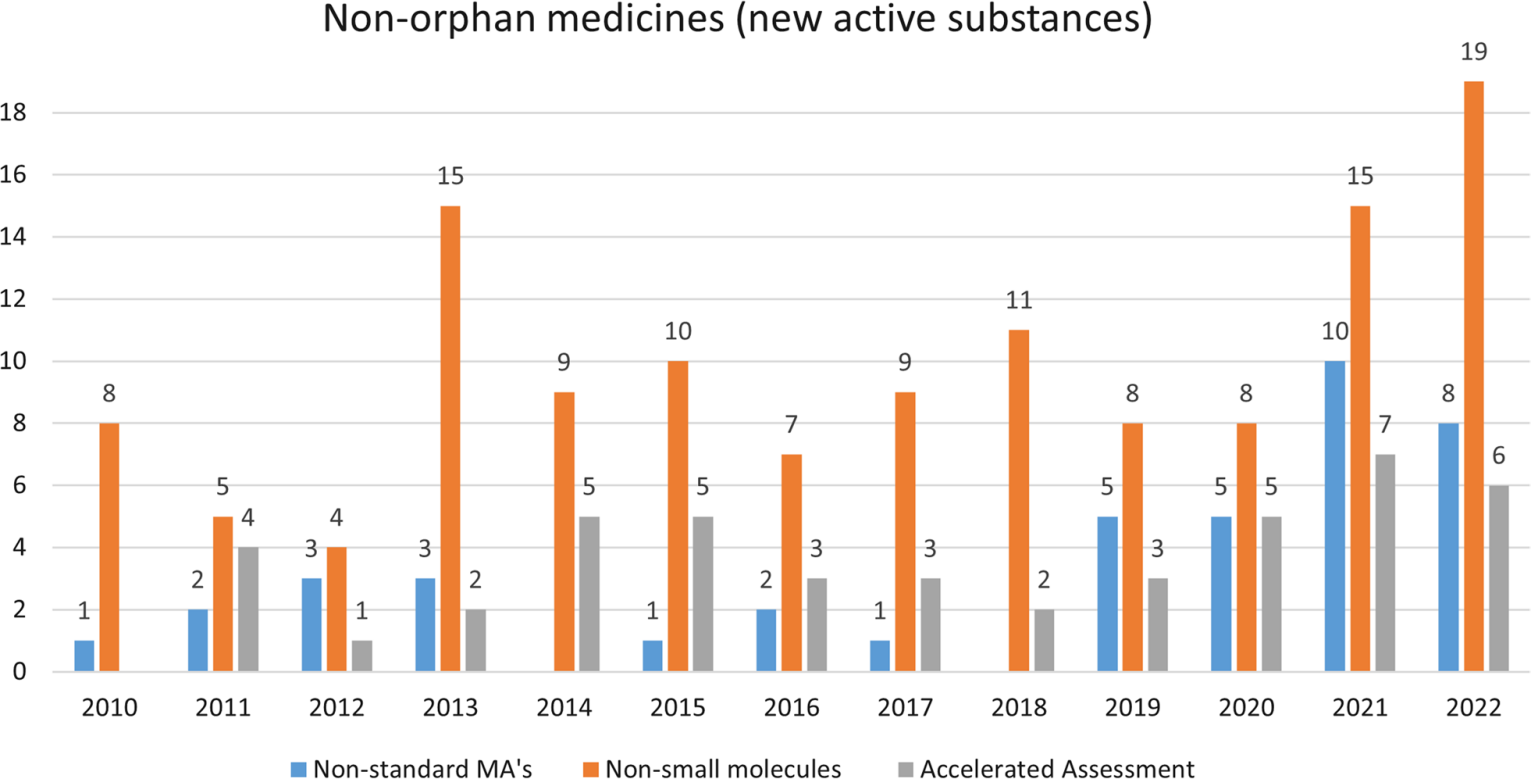
Evolution of the number of non-orphan products which have received a non-standard marketing authorization, non-orphan products which are non-small molecules, and non-orphan products which have benefited from accelerated assessment

About 24% (47/192) of the OMPs were eligible for accelerated assessment (Table 1) and 89% (42/47) of the MA’s which benefited from this accelerated assessment were granted from 2015 onwards (Fig. 2). Accelerated assessment is less frequent in case of non-orphan medicinal products. Figure 3 shows a.o. the evolution of the number of accelerated assessments granted to non-orphan products, over the period studied.

### Type of molecule

Biologicals, ATMP and oligonucleotides represent 45% (88/192) of the OMPs approved between 2010 and 2022 (Table 1).

The first ‘orphan’ Advanced Therapeutic Medicinal Product (ATMP) was approved in 2016 and this category constitutes 8% (15/192) of the OMPs approved in the period studied. The number of (orphan) non-small molecules (ATMP, biologicals and oligonucleotides) approved in each year is shown in Fig. 2.

The increasing trend in non-small molecules is less pronounced in the group of non-orphan medicinal products (Fig. 3). However, notable is that 30 of the 128 (23%) of the non-small molecules in the group of non-orphan medicines are vaccines. In the group of OMPs we did not identify any vaccine with orphan indications in the period studied.

### Repurposing

About 18% (33/192) of the OMPs approved were “old” molecules (approved > 10 years) used already (or in the past) in clinical practice for another indication. These data indicate that approximately one of five OMPs is repurposed (Table 1). 31 of the 33 OMPs classified as repurposed molecules are small molecules, one herbal preparation and one biological. Regarding the therapeutic indications, this group shows a high heterogeneity: about 20% has oncological indications, 20% is intended to treat metabolic disorders and the other 60% are a mix of different ATC codes and therapeutic areas.

### ATC code and therapeutic area

The OMPs identified belong to 14 different ATC first-level categories (Table 1). The ATC code L (Antineoplastic and immunomodulating agents) represents the group with highest proportion (40%), followed by A (Alimentary tract and metabolism) representing 17% of the OMPs approved. Two OMPs approved in 2022 had no ATC code yet, at the time of the analysis (one intended to treat lymphoproliferative disorders and the other to treat haemophilia A).

In the oncological group (ATC code L—Antineoplastic and immunomodulating agents) we identify therapeutic areas for which more than one OMP are approved. Multiple myeloma, leukaemia and lymphomas are the therapeutic areas where we observe more competitiveness given that these are therapeutic areas known to be “crowded”. In the non-oncological group, the most common therapeutic areas are haemophilia (A and B), cystic fibrosis, muscular dystrophy and dystrophy, familial amyloidosis, and anaemias (including beta-thalassemia). There is one small group of anti-infective medicines (ATC code J and P) with multidrug-resistant tuberculosis being the most common therapeutic area in this group (Table 1).

### Disease prevalence and target age

The prevalence of the diseases intended to treat by the OMPs approved between 2010–2022 were categorized as represented in Table 1. More than 50% of the OMPs were intended to treat diseases with a prevalence lower than or equal to 1 in 10 000. Only 12 OMPs (6%) were approved to treat a disease with a prevalence superior to 4 in 10 000.

About 55% of the OMPs was approved only for administration in adults and 20% is intended to treat both adults and children.

### Type of MA

Non-standard MA’s, i.e. conditional and approval under exceptional circumstances, represent about one-third (32%) of the OMPs between 2010–2022 (Table 1).

We observe an increase through this period, with only 5 of the 19 (26%) non-standard MA’s issued between 2010–12 and 27 of the 68 (40%) non-standard MA’s being issued between 2020–22 (Fig. 2). Non-standard MA’s are less frequent in the non-orphan medicines group but, as in the orphan medicines group, we observe an increasing trend of this type of MA’s over the period studied (Fig. 3).

Ninety four percent (94%) of the non-standard MA’s granted to OMPs were applications under article 8.3—full application—of Directive 2001/83 (EC). In the period studied 39 of the 40 conditional MA’s granted were for applications under legal basis 8.3 of Directive 2001/83 (EC). 65% of the conditional MA’s are for products with ATC code L (antineoplastic and immunomodulating agents) and mainly to treat adults. Only 2 of the 40 conditional approvals are repurposed molecules. A total of 23 out of the 41 (56%) OMPs with a conditional approval are non-small molecules. 15 of the 22 (68%) OMPs approved under exceptional circumstances between 2010 and 2022 are biologicals or ATMPs. The most prevalent ATC code in this group is the ATC code A—Alimentary tract and metabolism.

### Additional monitoring

About 124 of the 192 (65%) OMPs approved were added in the list of products under additional monitoring at the time of granting of the MA; 69 of the 124 (57%) of the OMPs under additional monitoring are standard MA’s. 75 of the 124 (60%) OMPs under additional monitoring are non-small molecules. 25 of the 68 (37%) OMPs not included in the additional monitoring list are repurposed molecules.

### Number of pivotal efficacy clinical trials for each indication approved

The range of main clinical trials supporting the indication approved varies between 0 (zero) and five (Table 1). Additionally, 135 of the 192 (70%) MA’s granted in this period were approved based on only one pivotal clinical trial. Ten OMPs were approved without presenting new clinical data. All these 10 OMPs were submitted under legal basis Article 10(a)—well-established use or 10.3—hybrid application of Directive 2001/83 (EC). We identified one OMP (fosdenopterin), approved in 2022, for which 5 main clinical studies were conducted to support the indication: molybdenum-cofactor-deficiency (MoCD) type A. From these 5 main studies, three are non-interventional studies (one is a retrospective observational data collection study, one is a natural history study and other is a follow-up data collection study).

Three OMPs have been approved based on clinical data of only ten patients. One is an ATMP intended to treat Aromatic L-amino acid decarboxylase deficiency. The other two are small molecules (glibenclamide to treat neonatal diabetes mellitus and setmelanotide to treat obesity associated with biallelic pro-opiomelanocortin (POMC), including PCSK1, deficiency obesity or leptin receptor (LEPR) deficiency obesity). All these conditions are ultra-rare (prevalence inferior to 0.2 per 10 000).

All ATMPs and all 6 oligonucleotides were approved based on only one pivotal clinical trial.

### Clinical trial designs

241 main studies were submitted to support the approval of the 192 OMPs. Among main studies, 117 (48.5%) were randomized controlled, double-blind trials. In 95 of these 117 (81%) randomized controlled trials the placebo was used as control whereas in 13 (11%) an active control was used. 91 OMPs (47%) were approved based on at least one randomized controlled trial. 4 OMPs were approved based on only retrospective/ observational studies. One of these OMPs was a repurposed molecule (Chenodeoxycholic Acid) and the retrospective study submitted was a cohort to assess safety and efficacy. Two biologicals were approved also based on retrospective studies: the applicant of “ex vivo expanded autologous human corneal epithelial cells containing stem cells” used a retrospective, uncontrolled, case series-based observational study and “dinutuximab beta” was approved based on two retrospective data collection studies with comparison to historical controls from a Patient Registry. Lonafarnib, to treat progeria, presented an observation cohort survival study.

### Type of primary efficacy endpoints

We did not include in this analysis the 11 OMPs which did not present new clinical data and, therefore, do not have endpoints to be classified. In the group of oncological OMPs, only 25% of the OMPs were approved based on hard clinical endpoints (“death” or “overall survival”). The most common efficacy endpoints used in this group are the surrogate endpoints (Tabel 1), such as: objective response rate (ORR), progression-free survival (PFS), disease-free survival (DFS), event-free survival (EFS) and pathological complete response (pCR). In the group of the OMPs addressing genetic diseases the clinical endpoints are the most common efficacy endpoints used (about 40%). In this group efficacy endpoints such as: “number of bleeding episodes”, “bleeding requiring blood transfusion” and “hospitalization required” are quite common and were used as clinical endpoints to assess the efficacy of the treatment of Haemophilia A and B. 28 of the 109 (26%) OMPs addressing genetic diseases have as primary endpoints the performance in disease scores (functional endpoints) such as: “change in 6-min walk distance (6MWD)”, “change in neuropathy impairment score plus 7 nerve test (mNIS + 7)”, “3-min stair climb test (3MSCT)” and “total motor function measures 32 score (MFM32)”. For cystic fibrosis all studies used “change in forced expiratory volume (FEV)”. In the small group of anti-infective OMPs, to assess the efficacy in the tuberculosis, only the following surrogate endpoints were used: “time to culture conversion” and “bacteriologic relapse/failure”.

### Total number of patients enrolled in the pivotal efficacy studies

For the calculation of the statistical parameters, we excluded the outlier 6886 (Malaria studies: SEAQUAMAT and AQUAMAT). Excluding this, the second maximum identified (1445) was from a OMP (tolvaptan), intended to treat autosomal dominant polycystic kidney disease, a condition with a prevalence reported of 4 in 10 000.

The minimum of patients enrolled in the pivotal clinical trial was 10. The median and the mean was 166 and 245 respectively. If we consider only the OMPs intended to treat ultra-rare diseases the mean of the total patients included in the main studies falls from 245 to 91.

The mean of number of patients enrolled in the oncological OMPs was calculated in 312; for the group of OMPs addressing genetic diseases was 207 and in the “old” molecules 494.

**Fig. 4:**
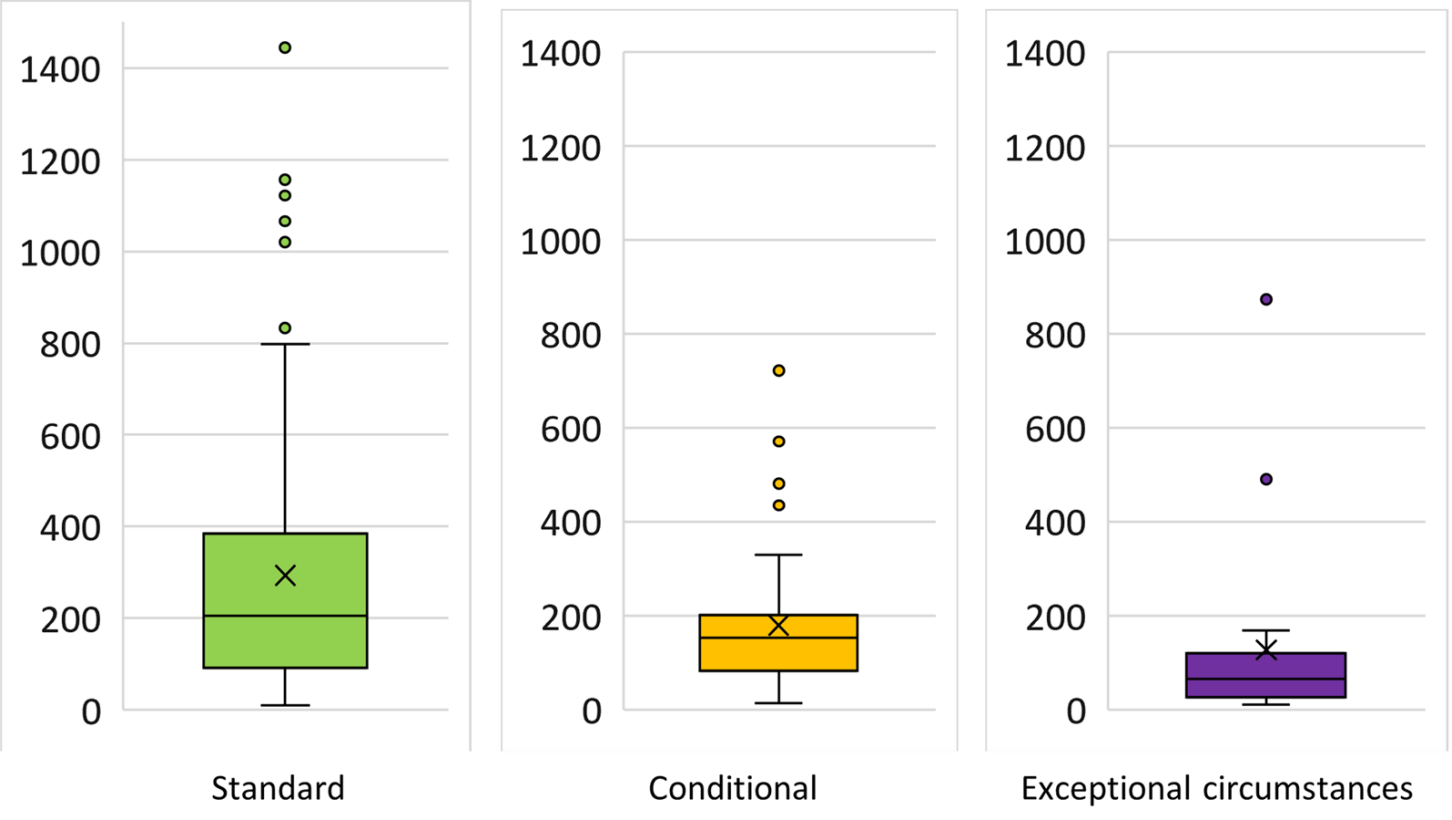
Box plot of the number of patients enrolled in the main clinical trials of OMP approved according to the type of MA granted between 2010–2022

In the next figure (Fig. 4) we present the boxplots of the total number of patients enrolled in the efficacy studies according to the type of MA granted.

## Discussion

A total of 192 OMPs were approved between 2010 and 2022. Overall, three main groups of OMPs were identified depending on their therapeutic indications: i) medicines with oncological indications, intended to treat adults (about 40% have oncological indications (ATC code L); ii) OMPs with ‘true’ rare indications, such as genetic and metabolic diseases [metabolic inborn errors, blood disorders, muscular dystrophies, and neuropathy’s (ATC codes A, B, M and N)], intended to treat mainly children and often approved based in only one pivotal clinical trial and iii) (small) group of OMPs is intended to treat infections (e.g. as multidrug-resistant tuberculosis, and other respiratory tract infections, mycosis fungoides, anthrax and malaria (ATC codes J and P).

The percentage of OMPs targeting oncological conditions (40%) identified in our study is similar to the results reported in a study analysing MA of OMPs between 2000 and 2013 (39%) [16]. Except for the seven major tumour types, including breast cancer in female, lung, colorectal, stomach, pancreatic, prostate and bladder cancers, almost all other cancers may be classified as rare [17, 18]. Applying the European definition of rare diseases (prevalence < 5 in 10 000), rare cancers represent 24% of total cancer prevalence as estimated by RARECARE [18]. Moreover, recent technical advances in genetic testing allowed further segmentation of cancer types into smaller subgroups [17, 19–22]. We observe in this category of OMPs a high level of competition which is not common in other orphan therapeutic areas: for some oncological therapeutic indications (e.g., multiple myeloma, leukaemia, and lymphoma) several OMPs were approved in the period studied. This high competition can be due to the high costs of the oncological medicines and the high probability of multiple indications, which makes this market attractive [23–26].

The majority (50–75%) of all rare diseases affect children and 30% of whom will die before age 5 years [27, 28]. We expected to see this reflected in the target age of the OMPs approved between 2010 and 2022. However, in the period studied, 55% of the OMPs approved were intended to treat adults only. This is due to the high number of OMPs approved with oncologic indications which are mainly to be used in adults. This confirms that several rare diseases remain without treatment, especially the non-oncologic diseases, as previously reported in existent literature [29–32].

An important criterion to classify a medicine as “orphan” is the prevalence of the disease that the medicine is intended to treat. The applicants usually present prevalence calculations based on publications derived from literature search. The EPARs and the public summaries of opinion on orphan designation always contain a section presenting the prevalence data. From 2018 onwards, for each newly authorised medicine, the COMP performs a review of the orphan designation based on the data available at the time of the MA application. This report (OMAR) is publicly available on the EMA website. In the period studied, most of the OMPs were intended to treat diseases with a prevalence lower than or equal to 1 in 10 000 (51%). Although no legal definition of ‘ultra-orphan’ diseases has been established, this subcategory was introduced by the National Institute for Health and Care Excellence (NICE). It is suggested to be applied to diseases with an estimated prevalence of < 1: 50 000 (or 0.2 in 10 000) [33]. In the period studied 41 of the 192 (14%) OMPs were intended to treat ultra-rare diseases. Some discrepancies were found with different prevalence figures reported for the same applied orphan indication. For instance, for multiple myeloma, 12 OMPs were approved in the period studied and the prevalence mentioned for this disease varies between 1.6 and 4 per 10 000. This discrepancy is, in some cases, difficult to explain since some of these orphan designations were granted in the same year. For panobinostat and elotuzumab, both intended to treat Multiple Myeloma, the orphan designation was granted in both cases in 2012. For panobinostat the prevalence reported was twice as high as the prevalence reported by the applicant of elotuzumab (3.2 vs 1.6 in 10 000). We would expect that for the same orphan designation granted in the same year, the prevalence calculated would be the same or, at least, similar. These discrepancies raise doubts regarding the source of information, validity and quality control check of the prevalence data provided by the applicant. We observed that since the introduction, in 2018, of the parallel assessment of the orphan designation criteria by the COMP, these discrepancies are not observed any longer. This additional step has contributed to a more precise, transparent, and efficient process in orphan medicines criteria assessment.

As expected, the majority of the orphan medicines received the status ‘new active substance’ and were approved under legal basis 8.3 of Directive 2001/83 (EC), i.e. they are full applications. Nevertheless, there is an interesting group of orphan medicines which represents ‘old molecules’ with new indications. This is called ‘drug repurposing’, ‘drug reprofiling’, ‘drug redirecting’ and/or ‘drug rediscovery’ [34]. In this study we adopted the term ‘drug repurposing’. The repurposed molecules identified between 2010 and 2022 were approved under legal basis 10(a), 10.3 or 8.3 of Directive 2001/83 (EC). The major advantage of drug repurposing is the availability of clinical and regulatory knowledge on the active substance’s safety profile, pharmacokinetics, dose, quality, and production process, hence typically lowering overall risk and development costs [35–37]. Drug repurposing may be particularly attractive for the development of treatments for rare diseases [35, 38]. The process of repurposing drugs for new indications, compared with the development of novel orphan drugs, is a time-saving and cost-efficient method resulting in higher success rates, which can therefore drastically reduce the risk of drug development for rare diseases [37, 39]. It can be expected that the new computational tools developed and made possible thanks to the artificial intelligence will contribute to this drug rediscovery process for rare diseases in a more efficient way [37, 40]. In the period studied about one out of five OMPs has been repurposed, which is similar with the findings reported by *Davies *et al. [41] and *Sibren van den Berg *et al. [36], both referring to the European setting. There is also one OMP—artesunate—approved in 2021, to treat severe malaria, with the status of new active substance, which is actually not ‘new’ since artesunate is being used for malaria treatment since 2006 [42–45]. Artesunate was available in the EU, in many treatment centers, since 2007, as non-licensed product [45–47]. Since artesunate is not a constituent of a medicinal product previously authorised within the EU, the ‘new active substance status’ was granted. Thus, this ‘old’ molecule could not be classified as ‘repurposed’.

The accelerated assessment, created in 2004, by the recital 33 and Article 14(9) of Regulation (EC) No 726/2004, reduces the timeframe for the EMA to review a marketing authorization application (MAA) [48]. Under the PRIME scheme, launched in 2016, it is possible for the applicants to receive confirmation during the clinical development phase that the investigational product might potentially be eligible for accelerated assessment [49]. Although, the PRIME program and the accelerated assessment tool are not exclusive for orphan medicines, the OMPs constituted 56% of the PRIME applications, according to the 5-years PRIME report [50]. During the period studied 89% of the MA’s which benefited from this accelerated assessment were granted from 2015 onwards, which was expected since PRIME was created in 2016.

After the assessment of the MAA, one of the three types of Marketing Authorizations can be granted: standard MA (valid for five years), conditional MA or MA under exceptional circumstances. The legal basis for the conditional MA is the Article 14 (a) of the Regulation (EC) No 726/2004 [48], and the provisions for granting a conditional MA are further elaborated in Regulation (EC) No 507/2006 [51]. A conditional MA is a pragmatic tool for the fast-track approval of a medicine that fulfils an unmet medical need.

The EC may grant a conditional MA for medicines that meet the following criteria: the benefit-risk balance of the medicine is positive; it is likely that the applicant will be able to provide comprehensive data post-authorization; the medicine fulfils an unmet medical need; the benefit of the medicine's immediate availability to patients is greater than the risk inherent in the fact that additional data are still required [52]. This type of MA is valid for one year and can be renewed annually. The Marketing Authorization Holder (MAH) must fulfil specific obligations such as completing on-going studies or perform new studies or collect additional data to confirm that the medicine’s benefit-risk balance remains positive. It is possible that the conditional MA becomes a standard MA, no longer subject to specific obligations, once the MAH fulfils the commitments imposed and the complete data have confirmed that the benefits continue to outweigh the risks [53]. Medicines for human use are eligible if they are intended for treating, preventing, or diagnosing seriously debilitating or life-threatening diseases [52]. This includes orphan medicines.. Since this special type of MA is the second most prevalent type of MA’s in the OMPs, it would be interesting to examine the clinical data/evidence supporting the MA, the reasons for granting the conditional authorization and to follow up the specific obligations imposed by the EMA. *Banzi *et al. assessed the conditional approvals between 2006 and 2015 [54]. However, their study did not specify the OMPs, and it did not include the period after PRIME implementation. Moreover, it is expected that the RWD and RWE will be essential for the evidence generation for this group of OMPs. Therefore, it would be interesting also to investigate and describe the role of the RWE for the orphan medicines development and regulatory decision making, for the same period, in further research.

On the other hand, the MA under exceptional circumstances, mentioned in the Article 14 [8] of the Regulation (EC) No 726/2004 [48], can be granted when the applicant is unable to provide comprehensive data on the efficacy and safety under normal conditions of use, because the condition to be treated is rare or because collection of full information is not possible or is unethical. Therefore, it will not lead to the completion of a full dossier and become a standard MA. The designated OMPs are eligible for approval under exceptional circumstances if the criteria considered for the approval under exceptional circumstances are fulfilled [52, 53]. All medicines approved under exceptional circumstances are under additional monitoring, as expected, since the aim of this additional monitoring is to enhance reporting of suspected adverse drug reactions for medicines for which the clinical evidence base is less well developed. The concept of additional monitoring was introduced by the 2010 pharmacovigilance legislation, which came into effect in July 2012.

In the period studied we observed that the OMPs added in the additional monitoring list are quite heterogeneous: different legal basis of the MAA, all three types of MA granted, different types of molecules and different ATC codes, which can be explained by the different situations where the additional monitoring should be applied: if the product contains a new active substance authorised in the EU after 1 January 2011; if the product is a biological medicine, such as a vaccine or a medicine derived from plasma (blood), authorised in the EU after 1 January 2011; it has been given a conditional approval or approval under exceptional circumstances; when the MAH is required to carry out additional studies; it is authorised with specific obligations on the recording of suspected adverse drug reactions. Or when the Agency's Pharmacovigilance Risk Assessment Committee (PRAC) advises to place a medicine under additional monitoring. It is interesting to note that 8 of 33 (24%) of the ´orphan’ repurposed medicines, approved between 2010 and 2022, were added in the additional monitoring list. Five of 8 (62,5%) were included since they have received a conditional approval or an approval under special circumstances. Three of 8 (37,5%) OMPs under additional monitoring have received standard MA’s. It would be interesting to analyse the reasons for including these “old” and “well-known” molecules in the additional monitoring list. However, only one EPAR mentions the reason: a mandatory post-authorization safety study (PASS) registry to further characterise the long-term safety of the active substance regarding the important potential risks.

Even though orphan medicines can benefit of some level of regulatory flexibility, due to the inherent characteristics of rare diseases, the granting of a MA requires the same level of evidence [1]. However, generating robust evidence with a small sample of patients is a methodological and logistic challenge [55]. It is known that the MAAs for orphan medicines often include a lower number of pivotal clinical trials and less subjects enrolled [12, 56, 57]. In the period studied a high percentage (70%) of OMPs approved was based on only one ‘pivotal’ clinical trial.

It is also interesting to note that the only OMP whose approval was based on five main clinical trials (fosdenopterin) is intended to treat an ultra-rare disease, molybdenum-cofactor-deficiency (MoCD) and was approved under exceptional circumstances. In total 64 patients were enrolled.

The maximum number of patients in main clinical trials identified was 6886 for artesunate (Malaria studies: SEAQUAMAT and AQUAMAT). Malaria is an ultra-rare disease in EU, with a prevalence calculated in 0.12 per 10 000. However, it is not rare at all in other regions. In fact, malaria is endemic in 85 countries with 6 African (Sub-Saharan) countries contributing for 55% of all cases globally [58]. The patients included in both clinical studies mentioned above were recruited in Southeast Asia and Africa respectively, where malaria is endemic.

A common adaptation in the clinical development programs of orphan medicines is the use of surrogate endpoints, instead of clinical endpoints, to accelerate approval in case of rare, life-threatening diseases [16, 59–61]. In our study set it was possible to confirm this. In the period studied 51% of the pivotal clinical trials used surrogate endpoints to assess the efficacy of the experimental medicine. Similar results were found by *Hofer *et al. [62], based on a data set of all OMPs approved between 2000 to 2013. According to Hofer’s study, surrogate primary endpoints were used in 57% of the OMPs, if the applicant was a small enterprise, 52% if the applicant is a medium-sized company and 82% when the applicant´s company was a large company [62].

Regarding the (pivotal) clinical trial designs submitted about 49% of them are randomized controlled trials (RCT). This percentage of RCT is lower than the results of the first decade of OMPs [63]. *Joppi *et al. analyzed the 63 OMPs approved between 2000 and 2010. For these MAA were submitted 38 RCT (60%). Also, the percentage of placebo is quite different: 49% in the first decade and 81% between 2010 and 2022. Interestingly, the percentage of active control is the same in both studies (11%). We would expect a higher use of placebo in the first decade and a higher use of active control in our study period, since in the meanwhile there are for some conditions already an approved therapy. Therefore, we would expect to find higher percentages of active controls between 2010–2022 than in the first decade (2000–2010).

Another interesting and intriguing observation is the early removal of an ODD. This phenomenon raises many questions, especially when it happens upon request of the sponsor before the end of the market exclusivity period. There are different theories about the reasons for this early removal of the ODD. Montanaro et al., speculates about these premature withdrawals saying that probably, the earlier removal of the exclusivity of product A of a company would allow another company to enter the market with a similar product B without fulfilling the requirement of its superiority on product A [64]. Superiority can be difficult to assess in case of orphan medicines due to logistic and methodological challenges associated with the rarity of the disease. Or the second medicine (B) can have failed the superiority test. In both cases it is simpler for the applicant to negotiate with the first company (competitor) by offering a compensation to remove the ODD of the medicine already marketed for the same indication. These removals are observed particularly in antineoplastic medicines where there is a high market competition and where ‘medicines’ attempt to differentiate from each other.

The pharmaceutical regulation and all process involving medicines development and licensing have been more than ever under the attention and scrutiny of the society, especially since the COVID-19 pandemic. Under this scope, the need of increasing transparency in each step of the product lifecycle has been underlined by other researchers such as Montanaro et al. [64] in what concerns to the request of removal the orphan designation.

We foresee in the current context a good opportunity to reflect about the need of this requirement in the new pharma legislation in order to make the withdrawal process more transparent.

The commercial reasons that justify a medicine withdrawal are also an interesting point. From the data publicly available, it is not possible to know exactly what reasons are hidden under this umbrella term. The MAH is not obliged to give specific explanation about the reasons to withdraw a product if the argument is commercial. If there are no known safety concerns or lack of efficacy, all other reasons can be commercial or part of the company’s strategy.

The newly proposed pharmaceutical legislation will repeal the general pharmaceutical legislation and the legislation on paediatric medicines and on rare diseases. There will be consequences for the three categories of orphan medicines identified in this study, i.e. i) OMPs with oncological indications; ii) OMPs intended to treat genetic diseases, such as haemophilia, cystic fibrosis and muscular dystrophies and iii) OMPs to treat infections (e.g. multi-resistant drug tuberculosis). The major changes are related to the data protection and market exclusivity.

For repurposed medicines the data protection is proposed to be four years if they address an unmet medical need and if they demonstrate a significant clinical benefit. This might be an incentive to stimulate medicines repurposing addressing unmet medical needs [65]. Significant benefit it is defined in Regulation (EC) No 141/2000 as "a relevant advantage" or “major contribution to patient care”. This concept is unique in Europe, it does not exist in any other regulatory framework. It requires a new paradigm of adaptive pathways to generate evidence along the life cycle of a product. On the one hand it can be a challenge to assess significant benefit when meaningful data are lacking: in case of conditional approval, for instance. On the other hand, there are many opportunities to generate this evidence and to prove the existence of significant benefit for the patients. Some of these are: 1) post-marketing (re)assessment of the significant benefit, which is unfortunately not yet foreseen in the regulation, but would allow more flexibility without hampering the value of the orphan status; 2) promote the discussions on significant benefit and pro-actively raise questions on the evidence generation of significant benefit when the applicant seeks protocol assistance; 3) take into account the patient relevant outcomes measures / patient reported outcomes to support the claim of significant benefit and 4) Real World Data (RWD) can be a valuable tool to create the evidence needed in order to assess the real value and clinical benefit of the product in a real life setting.

The concept of ‘unmet medical need’ is an important requirement which will impact the orphan medicines market. We may expect that the designated orphan medicinal products shall be considered as addressing unmet medical needs. However, it will be necessary to create clear scientific guidelines on this issue.

Market exclusivity, one of the most relevant incentives created by the current orphan medicines Regulation, will decrease from ten (10) years to nine (9) years. There will be a possibility of extension. In total, a OMP can receive a maximum of 13 years market exclusivity. The proposed changes in the market exclusivity rules suggest that this exclusivity will be a reward based on specific conditions to be filled by the MAH, instead of something given at the time of the granting of the MA. For instance, if the product addresses an “high unmet medical need”, an extension of one year to the market exclusivity can be granted. Products are considered to address a high unmet medical need if: a) there is no authorised medicine for the condition, or the new product brings exceptional therapeutic advancement; and b) the new product results in a meaningful reduction in disease morbidity or mortality. This could be a challenge since one of the most common reasons for losing the ODD at the time of the MA is the lack of evidence that the product shows a significant benefit compared to the existing alternative. Moreover, most of the OMPs were approved based on the surrogate endpoints.

The OMPs based on bibliographic data i.e., legal basis 10(a)—well-established use—of Directive 2001/83 (EC), will receive five (5) years of market exclusivity instead of ten (10) years. This type of application might become less attractive in the future if the legislation come into force as it is currently projected. It is not expected that this approach will have a critical impact on the OMPs approved since according to our data set only 3% of the OMPs were approved under the legal basis article 10(a) of Directive 2001/83 (EC).

Apart from these foreseen challenges there will be also opportunities for the OMPs: the reduced EMA assessment time from 210 to 180 days and the EC approval within 46 days (instead of 67 days); the regulatory sandboxes for testing new regulatory approaches for novel technologies and the facilitating use of real-world evidence (RWE), as well as the innovative clinical trial designs, which will be especially useful for the clinical research for rare diseases.

Finally, it’s important to acknowledge that this study is subject to limitations. First, the study data are limited to OMPs approved between the period 2010 and 2022, excluding the first 10 years of the orphan drug regulation. However, we presented the most recent data about extensive characterisation of OMPs approved in EU over along but a recent period. Second, we only analysed the OMPs approved i.e., with a MA granted, excluding negative CHMP opinions and all other orphan drug designations granted but which are not approved yet. The SME status presented could be not the same at the time of the application. Thus, it is possible that at the time of the application, the applicant had a SME-status, but later has lost it for any reason (e.g., company growth). We could not trace if at the time of the application the applicant had a SME-status.

## Conclusions

The number of OMPs approved in each year in the European Union shows an increasing trend, in the period studied. We even observed that during the years of the COVID-19 Pandemic (2020–2022), which placed a sustained and intense demand on the EU Medicines Regulatory Network (EUMRNs) resources, with multiple medicinal products subjected to accelerated evaluation and safety monitoring of the COVID-treatments, the highest number of OMPs have been approved.

The largest group of OMPs approved have oncological therapeutic indications and are intended to treat adults. In this group we observe different OMPs approved for the same indication, revealing a high competition in some therapeutic areas.

This analysis of OMPs approved in EU between 2010 and 2022 shows that also a shift has occurred in the rare disease medicine development space. Over the period studied we observe an increase of non-small molecules approved, accelerated assessment received and non-standard MA’s granted.

## Data Availability

We used public and freely available data from European public assessment reports (EPARs) and orphan drug designation reports from the web page of the European Medicines Agency (EMA). Please contact the author for specific data requests.

## Abbreviations

ATMP: Advanced therapeutic medicinal product
CHMP: Committee for medicinal products for human use
COMP: Committee for orphan medicinal products
DFS: Disease-free survival
EFS: Event-free survival
EC: European Commission
EMA: European medicines agency
EPAR: European public assessment report
EU: European Union
EUMRN: EU medicines regulatory network
FEV: Forced expiratory volume
ITT: Intention-to-treat population
MAA: Marketing authorization application
MAH: Marketing authorization holder
MS: Member state
ODD: Orphan drug designation
OMAR: Orphan maintenance assessment report
OMP: Orphan medicinal product
ORR: Objective response rate
OS: Overall survival
pCR: Pathological complete response
PFS: Progress-free survival
PRAC: Pharmacovigilance Risk Assessment Committee
PRIME: Priority medicines
RCT: Randomized controlled trials
RWD: Real world data
RWE: Real world evidence
SME: Small and medium sized enterprises
US: United States

## References

[1] Regulation (EC) No 141/2000 of the European Parliament and of the Council of 16 December 1999 on orphan medicinal products. Official Journal of the European Union. 2000;L 18:1–5.

[2] Delaye J, Cacciatore P, Kole A. Valuing the “burden” and impact of rare diseases: a scoping review. Vol. 13, Frontiers in Pharmacology. Frontiers Media S.A.; 2022. 10.3389/fphar.2022.914338 PMC9213803 35754469

[3] Lopes-Júnior LC, Ferraz VEF, Lima RAG, Schuab SIPC, Pessanha RM, Luz GS, et al. Health policies for rare disease patients: a scoping review. Vol. 19, International Journal of Environmental Research and Public Health. MDPI; 2022. 10.3390/ijerph192215174 PMC9690117 36429893

[4] Neez E, Hwang T, Sahoo SA, Naci H (2020). European Medicines Agency’s Priority Medicines (PRIME) scheme at 2 years: an evaluation of clinical studies supporting eligible drugs. Clin Pharmacol Ther.

[5] Hanaizi Z, Kweder S, Thor S, Ribeiro S, Marcal A (2023). Considering global development? Insights from Applications for FDA breakthrough therapy and EMA PRIME designations. Ther Innov Regul Sci.

[6] Montserrat A, Taruscio D (2019). Policies and actions to tackle rare diseases at European level. Ann Ist Super Sanita.

[7] Montserrat Moliner A, Waligora J. The European Union policy in the field of rare diseases. In: Advances in Experimental Medicine and Biology. Springer New York LLC; 2017. p. 561–87. 10.1007/978-3-319-67144-4_30 29214592

[8] Moliner AM (2010). Creating a European union framework for actions in the field of rare diseases. Adv Exp Med Biol.

[9] Nagai S. Flexible and expedited regulatory review processes for innovative medicines and regenerative medical products in the US, the EU, and Japan. Vol. 20, International Journal of Molecular Sciences. MDPI AG;2019. 10.3390/ijms20153801 PMC6696404 31382625

[10] Darrow JJ, Avorn J, Kesselheim AS (2020). FDA approval and regulation of pharmaceuticals, 1983–2018. JAMA J Am Med Assoc.

[11] Pontes C, Fontanet JM, Vives R, Sancho A, Gómez-Valent M, Ríos J (2018). Evidence supporting regulatory-decision making on orphan medicinal products authorisation in Europe: methodological uncertainties. Orphanet J Rare Dis.

[12] Picavet E, Cassiman D, Hollak CE, Maertens JA, Simoens S (2013). Clinical evidence for orphan medicinal products—A cause for concern?. Orphanet J Rare Dis.

[13] Kimmel L, Conti RM, Volerman A, Chua KP (2020). Pediatric orphan drug indications: 2010–2018. Pediatrics.

[14] Giannuzzi V, Conte R, Landi A, Ottomano SA, Bonifazi D, Baiardi P, et al. Orphan medicinal products in Europe and United States to cover needs of patients with rare diseases: an increased common effort is to be foreseen. Orphanet J Rare Dis. 2017;12(1). 10.1186/s13023-017-0617-1 PMC5376695 28372595

[15] Langedijk J, Mantel-Teeuwisse AK, Slijkerman DS, Schutjens MHDB (2015). Drug repositioning and repurposing: terminology and definitions in literature. Drug Discov Today.

[16] Kakkis ED, O’Donovan M, Cox G, Hayes M, Goodsaid F, Tandon PK, et al. Recommendations for the development of rare disease drugs using the accelerated approval pathway and for qualifying biomarkers as primary endpoints. Orphanet J Rare Dis. 2015;10(1). 10.1186/s13023-014-0195-4 PMC4347559 25757705

[17] Korchagina D, Jaroslawski S, Jadot G, Toumi M. Orphan drugs in oncology. In: Recent results in cancer research. Springer New York LLC; 2019. p. 109–42. 10.1007/978-3-030-01207-6_8 30543010

[18] Gatta G, Van Der Zwan JM, Casali PG, Siesling S, Dei Tos AP, Kunkler I (2011). Rare cancers are not so rare: the rare cancer burden in Europe. Eur J Cancer.

[19] Zimmermann BM, Eichinger J, Baumgartner MR. A systematic review of moral reasons on orphan drug reimbursement. Vol. 16, Orphanet Journal of Rare Diseases. BioMed Central Ltd;2021. 10.1186/s13023-021-01925-y PMC8247078 34193232

[20] Ginsburg O, Ashton-Prolla P, Cantor A, Mariosa D, Brennan P. The role of genomics in global cancer prevention. Vol. 18, Nature Reviews Clinical Oncology. Nature Research; 2021. p. 116–28. 10.1038/s41571-020-0428-5 32973296

[21] Zhao L, Lee VHF, Ng MK, Yan H, Bijlsma MF (2019). Molecular subtyping of cancer: current status and moving toward clinical applications. Brief Bioinform.

[22] Gao F, Wang W, Tan M, Zhu L, Zhang Y, Fessler E, et al. DeepCC: a novel deep learning-based framework for cancer molecular subtype classification. Oncogenesis. 2019;8(9). 10.1038/s41389-019-0157-8 PMC6697729 31420533

[23] Li X, Kockaya G, Research E, Wang H, Ni Y. Using 5 consecutive years of NICE guidance to describe the characteristics and influencing factors on the economic evaluation of orphan oncology drugs [Internet]. Available from: https://www.nice.org.uk/10.3389/fpubh.2022.964040 PMC9519130 36187695

[24] Salas-Vega S, Shearer E, Mossialos E (2020). Relationship between costs and clinical benefits of new cancer medicines in Australia, France, the UK, and the US. Soc Sci Med.

[25] Falcone R, Lombardi P, Filetti M, Duranti S, Pietragalla A, Fabi A, et al. Oncologic Drugs Approval in Europe for Solid Tumors: Overview of the Last 6 Years. Cancers (Basel). 2022;14(4). 10.3390/cancers14040889 PMC8870299 35205637

[26] Mestre-Ferrandiz J, Towse A, Dellamano R, Pistollato M. Multi-indication pricing: pros, cons and applicability to the UK seminar briefing 56 multi-indication pricing: pros, cons and applicability to the UK [Internet]. 2015. Available from: https://www.researchgate.net/publication/292975383

[27] Wright CF, FitzPatrick DR, Firth H V. Paediatric genomics: diagnosing rare disease in children. Vol. 19, Nature Reviews Genetics. Nature Publishing Group;2018. p. 253–68. 10.1038/nrg.2017.116 29398702

[28] The Lancet Neurology. Rare diseases: maintaining momentum. Vol. 21, The Lancet Neurology. Elsevier Ltd; 2022;203. 10.1016/S1474-4422(22)00046-1 35182497

[29] Ferreira CR. The burden of rare diseases. Vol. 179, American Journal of Medical Genetics, Part A. Wiley-Liss Inc.; 2019. p. 885–92. 10.1002/ajmg.a.61124 30883013

[30] Lancet Diabetes T. Spotlight on rare diseases. Lancet Diabetes Endocrinol. 2019;7:75. 10.1016/S2213- 10.1016/S2213-8587(19)30006-3 30683214

[31] Dawkins HJS, Draghia-Akli R, Lasko P, Lau LPL, Jonker AH, Cutillo CM, et al. Progress in rare diseases research 2010–2016: an IRDiRC perspective. Vol. 11, Clinical and Translational Science. Blackwell Publishing Ltd; 2018. p. 11–20. 10.1111/cts.12501 PMC5759730 28796411

[32] EURORDIS Rare Diseases Europe. #30Million reasons—factsheet [Internet]. 2021 [cited 2022 Jul 13]. Available from: https://www.eurordis.org/publications/why-we-need-european-action-on-rare-disease/

[33] London. Nice citizens council report ultra orphan drugs. 2004. 28230958

[34] Langedijk J, Mantel-Teeuwisse AK, Slijkerman DS, Schutjens MHDB. Drug repositioning and repurposing: terminology and definitions in literature. Vol. 20, Drug Discovery Today. Elsevier Ltd; 2015. p. 1027–34. 10.1016/j.drudis.2015.05.001 25975957

[35] Fetro C, Scherman D (2020). Drug repurposing in rare diseases: myths and reality. Therapie.

[36] van den Berg S, de Visser S, Leufkens HGM, Hollak CEM (2021). Drug repurposing for rare diseases: a role for academia. Front Pharmacol.

[37] Roessler HI, Knoers NVAM, van Haelst MM, van Haaften G (2021). Drug repurposing for rare diseases. Trends Pharmacol Sci.

[38] Scherman D, Fetro C (2020). Drug repositioning for rare diseases: Knowledge-based success stories. Therapie.

[39] Cha Y, Erez T, Reynolds IJ, Kumar D, Ross J, Koytiger G (2018). Drug repurposing from the perspective of pharmaceutical companies. Br J Pharmacol.

[40] Levin JM, Oprea TI, Davidovich S, Clozel T, Overington JP, Vanhaelen Q, et al. Artificial intelligence, drug repurposing and peer review. Vol. 38, Nature Biotechnology. Nature Research; 2020. p. 1127–31. 10.1038/s41587-020-0686-x 32929264

[41] Davies EH, Fulton E, Brook D, Hughes DA (2017). Affordable orphan drugs: a role for not-for-profit organizations. Br J Clin Pharmacol.

[42] Roussel C, Caumes E, Thellier M, Ndour PA, Buffet PA, Jauréguiberry S (2017). Artesunate to treat severe malaria in travellers: Review of efficacy and safety and practical implications. J Trav Med.

[43] Rolling T, Agbenyega T, Krishna S, Kremsner PG, Cramer JP (2015). Delayed haemolysis after artesunate treatment of severe malaria—Review of the literature and perspective. Travel Med Infect Dis.

[44] Barradell LB, Fitton A, Arnold K, van Thiel P. Product Development Unit, World Health Organization Special Programme for Research and Training in Tropical Diseases; World Health Organization. Vol. 50, Drugs. S. Hoffman, Malaria Program; 1995.

[45] Leblanc C, Vasse C, Minodier P, Mornand P, Naudin J, Quinet B (2020). Management and prevention of imported malaria in children. Update of the French guidelines. Med Mal Infect.

[46] Lalloo DG, Shingadia D, Bell DJ, Beeching NJ, Whitty CJM, Chiodini PL (2016). UK malaria treatment guidelines 2016. J Infect.

[47] Kreeftmeijer-Vegter AR, Van Veldhuizen CKW, De Vries PJ. Roll out of intraveneous artesunate under named patient programmes in the Netherlands, Belgium and France. Orphanet J Rare Dis. 2013;8(1). 10.1186/1750-1172-8-150 PMC3848968 24063858

[48] Regulation (EC) No 726/2004 of the European Parliament and of the Council of 31 March 2004. Official Journal of the European Union. 2004;L 136/1.

[49] European Medicines Agency. Enhanced early dialogue to facilitate accelerated assessment of PRIority Medicines (PRIME). 2018;(May).

[50] EMA. PRIME: Analysis of the first 5 years ’ experience. 2021; Available from: https://www.ema.europa.eu/en/documents/report/prime-analysis-first-5-years-experience_en.pdf

[51] Commission Regulation (EC) No 507/2006 of 29 March 2006 on the conditional marketing authorisation for medicinal products for human use falling within the scope of Regulation (EC) No 726/2004 of the European Parliament and of the Council. Official Journal of the European Union. L 92/6.

[52] Pignatti F, Péan E. Regulatory and evidence requirements and the changing landscape in regulation for marketing authorisation. In: Recent Results in Cancer Research. Springer New York LLC; 2019. p. 169–87. 10.1007/978-3-030-01207-6_11 30543013

[53] Llinares J. A regulatory overview about rare diseases. Vol. 686, Advances in Experimental Medicine and Biology. 2010. p. 193–207. 10.1007/978-90-481-9485-8_12 20824447

[54] Banzi R, Gerardi C, Bertele’ V, Garattini S. Approvals of drugs with uncertain benefit-risk profiles in Europe. Eur J Intern Med. 2015;26(8):572–84. 10.1016/j.ejim.2015.08.008 26342723

[55] Blin O, Lefebvre MN, Rascol O, Micallef J (2020). Orphan drug clinical development. Therapie.

[56] Kesselheim AS, Myers JA, Avorn J (2011). Characteristics of clinical trials to support approval of orphan vs nonorphan drugs for cancer. JAMA J Am Med Assoc.

[57] Jonker AH, Mills A, Lau LPL, Ando Y, Baroldi P, Bretz F, Burman CF, Collignon O Et al. Small population clinical trials: challenges in the field of rare diseases. 2016;(July).

[58] WHO. World Malaria report 2021.

[59] Cox GF (2018). The art and science of choosing efficacy endpoints for rare disease clinical trials. Am J Med Genet A.

[60] Bax BE. Biomarkers in rare diseases. Vol. 22, International Journal of Molecular Sciences. MDPI; 2021. p. 1–4. 10.3390/ijms22020673 PMC7827027 33445477

[61] Aronson JK (2005). Biomarkers and surrogate endpoints. Br J Clin Pharmacol.

[62] Hofer MP, Hedman H, Mavris M, Koenig F, Vetter T, Posch M, et al. Marketing authorisation of orphan medicines in Europe from 2000 to 2013. Vol. 23, Drug Discovery Today. Elsevier Ltd; 2018. p. 424–33. 10.1016/j.drudis.2017.10.012 29074441

[63] Joppi R, Bertele’ V, Garattini S. Orphan drugs, orphan diseases. The first decade of orphan drug legislation in the EU. Eur J Clin Pharmacol. 2013;69(4):1009–24. 10.1007/s00228-012-1423-2 23090701

[64] Montanaro N, Bonaldo G, Motola D (2021). Removal of the EMA orphan designation upon request of the sponsor: Cui prodest?. Eur J Clin Pharmacol.

[65] Fregonese L, Greene L, Hofer M, Magrelli A, Naumann-Winter F, Larsson K, et al. Demonstrating significant benefit of orphan medicines: analysis of 15 years of experience in Europe. Vol. 23, Drug Discovery Today. Elsevier Ltd; 2018. p. 90–100. 10.1016/j.drudis.2017.09.010 29024805

